# Supplementary material for: Effect of very large body mass loss on energetics, mechanics and efficiency of walking in adults with obesity: mass‐driven versus behavioural adaptations
Source: J Physiol. 2021 Sep 28;600(4):979–96. doi: 10.1113/JP281710 (PMC9293213; doi:10.1113/JP281710)
Supplement: Supplementary file 3 — Supplementary Table S1: Participant characteristics (raw data). [file TJP-600-979-s003.pdf]

Subjects	Age (yr)			Height (cm)			Body mass (kg)			Lean Mass (kg)			Fat Mass (kg)			BMI (kg/m^2)			Android/Gynoid		
	Bsl	Post1	Post2	Bsl	Post1	Post2	Bsl	Post1	Post2	Bsl	Post1	Post2	Bsl	Post1	Post2	Bsl	Post1	Post2	Bsl	Post1	Post2
1	37.0	---	---	159.5	---	---	129.4	103.6	104.9	48.4	43.6	42.6	76.6	56.2	57.8	50.9	40.7	41.2	1.1	1.2	1.1
2	44.1	---	---	169.0	---	---	113.8	84.7	75.6	64.1	58.0	58.0	45.0	22.3	14.8	39.8	29.6	26.5	1.8	1.3	1.1
3	35.3	---	---	161.0	---	---	114.4	80.6	72.8	55.1	47.1	47.2	53.1	29.9	21.1	44.1	31.1	28.1	1.1	1.1	0.9
4	23.8	---	---	169.5	---	---	119.0	87.3	76.7	48.9	42.5	43.7	66.5	41.6	29.5	41.4	30.4	26.7	1.1	1.0	0.8
5	45.7	---	---	169.0	---	---	110.9	82.0	72.6	53.8	46.3	46.1	52.3	32.2	23.3	38.8	28.7	25.4	1.2	1.1	1.0
6	39.6	---	---	163.0	---	---	124.4	95.7	95.9	51.6	46.2	46.2	69.6	46.4	45.6	46.8	36.0	36.1	1.1	1.1	1.1
7	40.6	---	---	161.2	---	---	105.9	82.3	81.1	47.9	42.6	42.6	53.7	36.3	34.3	40.8	31.7	31.2	1.2	1.2	1.1
8	54.8	---	---	162.0	---	---	94.1	67.3	55.0	47.1	37.7	39.3	42.5	25.5	12.6	35.9	25.7	20.9	1.5	1.5	1.2
9	34.4	---	---	163.0	---	---	120.6	84.2	72.5	54.9	46.1		63.3	34.4		45.4	31.7		1.2	1.1	

Subjects	Fat body mass (% body mass)			Lower limb mass (kg)			Lean lower limb mass (kg)			Fat lower limb mass (kg)			Upper limb mass (kg)			Lean upper limb mass (kg)		
	Bsl	Post1	Post2	Bsl	Post1	Post2	Bsl	Post1	Post2	Bsl	Post1	Post2	Bsl	Post1	Post2	Bsl	Post1	Post2
1	59.2	54.2	55.1	46.0	26.9	37.8	18.7	12.7	16.0	26.5	13.6	21.1	13.4	6.2	10.3	5.2	2.9	4.5
2	39.6	26.4	19.6	33.1	26.5	24.3	22.9	19.2	18.7	9.0	6.1	4.4	12.7	10.0	8.7	8.2	7.1	6.7
3	46.5	37.1	29.0	37.1	27.1	25.0	19.4	16.0	16.0	16.8	10.2	8.1	10.6	9.2	7.6	6.0	5.4	5.1
4	55.9	47.7	38.4	48.7	35.9	31.0	19.4	15.9	16.4	28.5	19.1	13.8	14.1	9.0	7.8	5.8	4.2	4.2
5	47.2	39.2	32.0	42.0	30.9	27.3	21.7	18.0	17.2	19.4	12.0	9.2	10.8	8.1	7.3	5.7	4.6	4.5
6	55.9	48.5	47.5	43.1	34.0	33.3	19.4	17.2	16.8	22.6	15.9	15.5	12.8	11.9	11.6	5.1	5.0	5.0
7	50.7	44.0	42.3	33.4	26.6	26.6	17.3	15.0	15.2	15.3	10.7	10.6	11.7	9.2	8.5	5.4	4.6	4.2
8	45.2	37.9	23.0	28.0	20.8	18.0	17.5	13.4	13.3	9.6	6.5	3.8	11.4	7.4	6.1	5.6	3.9	4.0
9	52.4	40.9		44.1	31.3		21.6	17.1		21.4	13.1		11.30	8.20		5.9	4.8	

Subjects	Fat upper limb mass (kg)			Trunk mass (kg)			Lean trunk mass (kg)			Fat trunk mass (kg)			VAT vol (cm3)			Physical activity level (score)		
	Bsl	Post1	Post2	Bsl	Post1	Post2	Bsl	Post1	Post2	Bsl	Post1	Post2	Bsl	Post1	Post2	Bsl	Post1	Post2
1	8.0	3.1	5.6	67.9	68.0	67.8	24.5	28.0	22.1	42.2	39.4	31.1	2.6	1.5	1.2	5.4	6.5	6.1
2	4.1	2.4	1.5	63.9	43.4	40.0	33.0	31.8	32.6	32.0	13.8	8.8	5.4	1.2	1.1	8.0	8.5	9.1
3	4.2	3.4	2.2	59.3	41.9	35.5	29.7	25.7	26.1	32.1	16.2	10.9	2.3	0.9	0.5	8.6	7.5	8.0
4	8.0	4.6	3.3	52.3	40.1	33.8	23.7	22.5	23.1	30.0	17.9	12.4	1.6	0.9	0.4	5.5	5.0	6.8
5	4.7	3.2	2.4	52.7	39.8	33.9	26.4	23.7	24.4	28.2	17.0	11.6	1.1	0.5	0.2	9.0	8.9	9.8
6	7.4	6.5	6.2	64.3	48.6	48.1	27.0	24.0	24.3	39.6	24.0	23.9	3.4	1.8	1.5	8.0	8.1	7.5
7	6.0	4.3	3.9	57.1	42.4	42.4	25.2	22.9	23.2	32.5	21.2	19.7	2.2	1.1	0.9	6.6	7.6	8.5
8	5.5	3.2	1.8	49.7	35.9	28.5	24.0	20.4	22.0	27.5	15.8	7.1	2.6	1.2	0.7	8.9	8.6	9.1
9	5.1	3.0		63.6	41.2		27.5	24.1		36.8	18.3		2.0	0.9		9.3	9.4	7.6
