# Supplementary material for: Effect of very large body mass loss on energetics, mechanics and efficiency of walking in adults with obesity: mass‐driven versus behavioural adaptations
Source: J Physiol. 2021 Sep 28;600(4):979–96. doi: 10.1113/JP281710 (PMC9293213; doi:10.1113/JP281710)
Supplement: Supplementary file 4 — Supplementary Table S2: Standing metabolic rate and energetics of walking (raw data). [file TJP-600-979-s007.pdf]

Subjects	RMR (W)			NCw (J/m)														
	Bsl	Post1	Post2	Bsl_V2	Bsl_V3	Bsl_V4	Bsl_V5	Bsl_V6	Post1_V2	Post1_V3	Post1_V4	Post1_V5	Post1_V6	Post2_V2	Post2_V3	Post2_V4	Post2_V5	Post2_V6
1	151.2	119.6	119.5	367.0	283.2	306.8	359.1		260.1	225.8	238.8	311.2		247.5	210.5	235.6	288.2	
2	147.4	134.2	106.2	302.4	279.0	269.8	306.5	396.8	254.0	239.5	221.9	225.0	276.5	235.5	195.7	192.9	218.1	252.6
3	127.8	92.6	110.2	267.4	302.5	311.1	307.4		188.8	187.0	213.4	240.2		196.7	132.4	147.3	159.0	211.3
4	117.0	107.1	102.1	325.8	259.3	256.8	265.5		282.8	208.3	203.9	229.3		200.4	183.5	165.8	187.7	
5	129.4	96.1	120.1	287.8	239.0	264.1	280.2	369.3	215.3	189.7	222.5	214.3	266.5	187.0	164.9	174.6	201.3	205.5
6	152.1	72.8	120.1	469.5	403.9	363.1	385.2	479.7	424.8	318.6	319.2	281.2	345.5	305.6	220.1	201.6	221.2	280.1
7	129.6	89.0	85.3	255.2	266.2	258.4	319.2	366.8	232.6	209.6	218.3	226.8	310.8	197.6	182.0	171.9	211.6	290.6
8	111.4	75.6	82.4	328.9	259.2	223.2	229.0		248.6	195.1	188.4	205.1	229.0	163.8	143.3	138.5	145.1	182.4
9	116.9	96.6	123.8	266.3	310.9	292.5	335.2	383.3	193.3	160.9	167.0	200.6	245.8	154.1	181.1	167.2	164.8	213.6
