# Supplementary material for: Effect of very large body mass loss on energetics, mechanics and efficiency of walking in adults with obesity: mass‐driven versus behavioural adaptations
Source: J Physiol. 2021 Sep 28;600(4):979–96. doi: 10.1113/JP281710 (PMC9293213; doi:10.1113/JP281710)
Supplement: Supplementary file 7 — Supplementary Table S5: Mechanics and mechanical efficiency (raw data). [file TJP-600-979-s001.pdf]

Subjects	Wext (J/m)														
	Bsl_V2	Bsl_V3	Bsl_V4	Bsl_V5	Bsl_V6	Post1_V2	Post1_V3	Post1_V4	Post1_V5	Post1_V6	Post2_V2	Post2_V3	Post2_V4	Post2_V5	Post2_V6
1	36.05	34.31	41.69	33.67		32.00	33.21	30.23	27.65		36.20	34.05	29.89	25.07	29.65
2	28.77	30.12	32.29	39.28	45.28	22.99	22.54	27.13	31.40	33.94	19.29	19.13	23.06	27.64	30.94
3	29.66	29.82	31.01	35.19		20.70	23.75	27.09	32.35		20.86	23.27	24.39	28.77	32.93
4	32.31	33.14	36.99	31.72		26.11	24.33	24.29	24.69		18.92	21.68	26.37	28.44	
5	25.44	26.01	25.03	31.45	40.35	19.15	17.00	20.80	29.22	37.36	16.22	16.38	17.49	25.26	34.78
6	34.57	35.65	36.19	34.58	41.99	24.71	25.52	26.09	27.52	27.39	24.11	26.31	23.43	25.33	27.91
7	30.38	30.50	30.00	36.53	44.46	20.84	21.70	24.22	27.55	34.44	19.23	21.44	24.65	27.69	32.27
8	28.78	30.60	34.93	37.06		21.45	24.23	26.99	30.99	32.89	18.72	19.46	23.27	25.93	28.88
9	34.96	32.18	33.69	35.17	39.63	24.01	22.39	27.27	30.25	35.85	19.10	18.84	20.57	25.85	33.49

Subjects	Wint (J/m)														
	Bsl_V2	Bsl_V3	Bsl_V4	Bsl_V5	Bsl_V6	Post1_V2	Post1_V3	Post1_V4	Post1_V5	Post1_V6	Post2_V2	Post2_V3	Post2_V4	Post2_V5	Post2_V6
1	30.94	34.75	40.62	49.64		26.29	22.96	26.16	35.80		22.33	21.07	24.03	35.01	49.87
2	18.62	22.12	25.61	43.23	46.35	14.07	16.55	19.97	25.47	28.15	13.40	14.64	17.73	19.93	23.24
3	29.91	28.09	29.43	38.22		18.19	19.02	20.18	27.31		15.80	14.98	17.67	20.75	25.96
4	27.83	29.82	36.28	44.89		29.23	25.00	28.93	36.66		15.58	19.02	23.52	27.91	
5	33.52	25.53	28.31	32.31	40.39	15.51	15.65	19.81	23.60	29.64	13.06	14.81	18.69	21.49	28.01
6	21.83	37.48	51.24	52.88	49.22	21.43	21.68	24.77	27.94	33.40	23.21	22.93	24.34	28.02	33.13
7	37.45	30.50	31.07	34.15	43.40	21.75	20.47	20.19	23.47	28.82	20.55	18.30	21.71	24.71	27.76
8	16.68	17.25	21.03	26.27		9.01	11.07	14.20	18.46	23.43	7.29	9.09	12.06	16.80	19.01
9	41.19	34.03	37.04	37.94	53.41	22.30	20.77	23.00	28.80	36.64					

Subjects	Wtot (J/m)														
	Bsl_V2	Bsl_V3	Bsl_V4	Bsl_V5	Bsl_V6	Post1_V2	Post1_V3	Post1_V4	Post1_V5	Post1_V6	Post2_V2	Post2_V3	Post2_V4	Post2_V5	Post2_V6
1	66.99	69.06	82.31	83.31		58.29	56.16	56.40	63.45		58.53	55.11	53.92	60.08	79.53
2	47.39	52.24	57.91	82.50	91.63	37.06	39.09	47.10	56.87	62.10	32.68	33.77	40.79	47.57	54.18
3	59.57	57.92	60.43	73.40		38.88	42.77	47.27	59.67		36.67	38.25	42.07	49.52	58.88
4	60.13	62.96	73.27	76.61		55.33	49.32	53.22	61.35		34.50	40.70	49.89	56.35	
5	58.96	51.54	53.34	63.76	80.74	34.66	32.65	40.61	52.82	67.00	29.29	31.18	36.18	46.75	62.79
6	56.40	73.13	87.43	87.46	91.21	46.14	47.20	50.85	55.46	60.79	47.32	49.24	47.77	53.35	61.04
7	67.83	61.00	61.08	70.68	87.86	42.58	42.17	44.41	51.01	63.26	39.78	39.74	46.36	52.40	60.03
8	45.47	47.84	55.95	63.34		30.45	35.30	41.19	49.45	56.31	26.01	28.55	35.33	42.73	47.89
9	76.15	66.21	70.73	73.11	93.03	46.30	43.17	50.26	59.05	72.49					

Subjects	Wk+ (J/m)														
	Bsl_V2	Bsl_V3	Bsl_V4	Bsl_V5	Bsl_V6	Post1_V2	Post1_V3	Post1_V4	Post1_V5	Post1_V6	Post2_V2	Post2_V3	Post2_V4	Post2_V5	Post2_V6
1	31.42	41.41	53.87	61.00		24.94	33.69	45.33	50.69		24.78	35.97	44.69	50.50	56.47
2	31.66	45.36	57.46	69.70	77.10	25.39	34.53	43.30	52.22	59.45	20.87	27.50	37.46	46.57	53.04
3	31.73	42.05	53.56	63.88		20.68	30.92	40.63	49.80		19.40	28.02	37.73	45.12	52.53
4	35.26	47.56	60.63	72.56		24.33	33.78	41.06	50.32		21.41	30.57	39.94	49.83	
5	34.41	43.03	51.59	64.31	74.58	24.39	31.54	40.12	51.04	60.38	18.10	25.65	33.71	44.02	54.43
6	43.01	49.13	53.53	60.12	71.48	29.36	34.96	42.79	51.41	56.71	28.50	34.24	40.94	48.83	56.49
7	36.78	42.92	50.28	64.75	70.16	24.99	31.89	38.72	48.40	58.37	22.01	29.17	37.13	45.79	54.22
8	24.68	33.97	46.33	55.27		20.46	26.83	35.69	43.09	48.97	15.74	19.65	27.53	34.73	40.12
9	36.17	48.83	62.07	70.72	80.60	24.12	33.83	44.36	52.92	62.12	19.76	27.80	36.45	44.67	54.31

Subjects	Wp+ (J/m)														
	Bsl_V2	Bsl_V3	Bsl_V4	Bsl_V5	Bsl_V6	Post1_V2	Post1_V3	Post1_V4	Post1_V5	Post1_V6	Post2_V2	Post2_V3	Post2_V4	Post2_V5	Post2_V6
1	53.79	65.71	87.30	86.32		43.47	54.66	66.85	70.85		44.92	55.26	66.10	67.08	52.07
2	52.32	61.61	64.59	75.36	74.66	38.61	44.55	48.14	48.02	51.44	33.45	33.97	41.08	42.76	43.15
3	43.30	54.77	63.25	69.64		30.09	37.59	42.66	48.54		27.99	33.72	39.00	43.27	42.84
4	61.84	72.25	82.79	79.25		47.14	52.07	55.88	56.28		38.58	43.30	46.42	49.29	
5	50.47	61.84	63.17	68.37	67.78	34.33	38.67	39.74	43.10	48.53	27.56	35.28	36.39	39.03	42.32
6	61.74	79.43	82.34	81.88	94.23	47.66	56.80	59.76	61.41	66.52	49.46	56.27	56.68	60.26	63.16
7	51.68	52.12	55.05	57.94	60.54	35.36	38.13	37.75	40.36	44.51	33.80	36.70	37.62	39.58	41.97
8	38.68	33.99	44.95	44.54		34.31	33.45	34.67	36.93	34.34	25.46	25.71	29.04	30.50	27.54
9	55.36	69.00	75.22	75.34	77.06	37.50	42.44	46.17	46.07	47.54	32.18	37.44	37.65	37.49	39.11

Subjects	Recovery (%)														
	Bsl_V2	Bsl_V3	Bsl_V4	Bsl_V5	Bsl_V6	Post1_V2	Post1_V3	Post1_V4	Post1_V5	Post1_V6	Post2_V2	Post2_V3	Post2_V4	Post2_V5	Post2_V6
1	58.45	69.24	71.64	78.67		53.71	62.86	73.81	78.04		48.46	63.09	73.75	79.52	73.55
2	66.73	73.15	75.40	74.58	72.00	64.62	72.27	71.09	69.54	70.25	64.88	69.39	72.12	70.54	68.58
3	61.34	70.28	74.91	75.37		59.62	65.91	68.17	68.69		56.26	62.80	68.88	68.18	66.24
4	68.07	73.97	75.60	80.97		63.84	72.29	75.72	77.67		69.12	71.31	70.26	72.81	
5	70.68	75.99	79.09	77.36	72.81	67.86	76.46	74.69	69.83	66.65	64.87	73.70	75.70	70.43	64.90
6	68.44	73.48	74.75	77.14	76.27	68.30	72.85	75.31	76.49	78.69	69.58	71.56	76.73	77.61	77.63
7	66.66	69.15	72.91	72.18	66.98	65.89	69.54	69.04	69.77	67.45	65.87	68.01	67.74	68.35	67.33
8	54.89	55.59	62.58	63.74		61.24	60.30	62.30	61.95	61.21	54.89	57.52	59.36	60.82	57.93
9	62.42	74.15	77.12	77.68	76.69	61.75	71.87	71.22	71.09	69.08	63.55	71.60	72.98	69.36	65.04

Subjects	Mechanical efficiency (%)														
	Bsl_V2	Bsl_V3	Bsl_V4	Bsl_V5	Bsl_V6	Post1_V2	Post1_V3	Post1_V4	Post1_V5	Post1_V6	Post2_V2	Post2_V3	Post2_V4	Post2_V5	Post2_V6
1	18.25	24.38	26.83	23.20		22.40	24.87	23.61	20.38		23.64	26.18	22.88	20.84	22.53
2	15.67	18.72	21.46	26.91	23.09	14.60	16.33	21.23	25.29	22.47	13.89	17.26	21.16	21.83	21.46
3	22.28	19.15	19.42	23.88		20.56	22.83	22.11	24.80		19.83	28.89	28.55	31.13	27.86
4	18.41	24.22	28.46	28.78		19.22	22.34	25.73	26.53		17.21	22.18	30.07	30.01	
5	20.49	21.56	20.20	22.76	21.86	16.10	17.21	18.25	24.65	25.14	15.66	18.90	20.72	23.23	30.55
6	12.02	18.11	24.09	22.71	19.02	10.21	14.02	15.29	18.99	17.15	16.15	22.57	23.86	24.24	21.86
7	26.58	22.91	23.64	22.13	23.95	18.26	20.06	20.28	22.42	20.30	21.71	23.05	28.16	25.47	21.02
8	13.82	18.45	25.06	27.65		12.23	18.06	21.83	24.07	24.55	15.88	19.91	25.51	29.45	26.25
9	28.58	21.28	24.17	21.80	24.26	24.12	28.12	31.14	30.10	29.94					
